# Implementation of early warning, alert and response: An experience from the Marburg virus disease outbreak response in Kagera, Tanzania, March to May 2023

**DOI:** 10.1371/journal.pone.0325823

**Published:** 2025-06-20

**Authors:** Atuganile Musyani, Emmanuel Mwakapasa, Marcelina Mponela, Lusungu Ngailo, Irene Rabiel, Mwanakombo Khama, Eninka Mmbaga, Calvin Sindato, Mololo Noah, Werema Solomon, George Mrema, Danstan Ngenzi, George Kauki, Gerald Manasseh, Michael Mahande, Emmanuel Sarakikya, Wilhellmuss Mauka, Lameck Machumi, Jonathan Mcharo, Mikidadi Mtalika, Christer Kanyankore, Kokuhabwa Mukurasi, Medard Beyanga, Pius Horumpende, Michael Kiremeji, Erick Kinyenje, Emmanuel Mnkeni, Missana Yango, Faith Melchiory Kundy, Sibomana Leonard, Patrick Mwanahapa, Rita Mutayoba, Mary Mayige, Wangeci Gatei, Vida Mmbaga, Grace Magembe

**Affiliations:** 1 Amref Health Africa Tanzania, Dar es Salaam, Tanzania; 2 Ministry of Health (MoH), Dodoma, Tanzania; 3 Tanzania Country Office of the U.S. Centers for Disease Control and Prevention, Dar es Salaam, Tanzania; 4 National Institute for Medical Research (NIMR), Dar es Salaam, Tanzania; 5 Prime Minister’s Office (PMO), Dodoma, Tanzania; 6 World Health Organization (WHO), Tanzania Country Office, Dar es Salaam, Tanzania; 7 President’s Office, Regional Administration and Local Government (PO-RALG), Dodoma, Tanzania; 8 Management and Development for Health (MDH), Dodoma, Tanzania; 9 Tanzania Wildlife Research Institute (TAWIRI), Arusha, Tanzania; Virginia Commonwealth University, UNITED STATES OF AMERICA

## Abstract

**Introduction:**

Tanzania declared a Marburg Virus Disease (MVD) outbreak on March 21, 2023, reporting nine cases and six deaths (case fatality rate (CFR) 66.7%). Detection began when a Community Health Worker (CHW) reported unexplained illness via the electronic EBS (e-EBS) system, triggering a national outbreak response. This study documents the Early Warning, Alert and Response (EWAR) interventions carried out during the MVD outbreak response in the Kagera region to identify strengths and bottlenecks for strengthening future outbreak preparedness and response efforts.

**Method:**

We documented EWAR interventions using retrospective surveillance document review. MVD outbreak detection and reporting timeliness were compared with Tanzania’s EBS indicators and the 7-1-7 target. Surveillance interventions included additional staff deployment, equipment addition, and tool adoption. Community sensitization efforts utilized Swahili-translated informational cards to facilitate early detection and reporting of signals through multiple channels, including the 199-hotline number, EBS desk numbers and via e-EBS and verified using the standard case definition (SCD). Signals were compiled in Microsoft Excel, where descriptive analysis using frequencies to show trends was conducted. Suspected MVD cases were sent for laboratory confirmation.

**Findings:**

On March 15, 2023, a CHW reported a signal in the e-EBS system within 24 hours. However, a community member and HCWs missed unusual signs of the MVD index case. Five additional members were deployed to support data management using the equipment provided, including three laptops, ten smartphones, and adapted tools. A total of 6,260 informational cards were distributed during community sensitization; 176 MVD signals were reported, where 48 (27.3%) met the SCD, and 37 were sent for laboratory confirmation, of which 2.7% tested positive for the virus. Most signals, 107 (60.8%), were reported in April.

**Conclusions and recommendations:**

The government should adopt the 7-1-7 target and strengthen community and health facility EBS through ongoing mentorship for EWAR.

## Introduction

The Tanzania Ministry of Health (MoH) declared the outbreak of Marburg Virus Disease (MVD) on March 21, 2023 [1]. The outbreak occurred in the Bukoba district of the Kagera region, northwest of the country [2]. By the end of the outbreak, nine (9) cases, of which eight (8) were confirmed and one probable, were reported, with six (6) deaths (case fatality rate (CFR) of 66.7%). MVD is a highly lethal hemorrhagic fever caused by the Marburg virus, a member of the Filoviridae family, which also includes the Ebola Virus Disease (EVD) [3–5]. The mortality rate for MVD varies widely, from 32% to 88%, with an incubation period of 2–21 days [3–7].

The Tanzania MVD outbreak in the Kagera region was the 16^th^ recorded MVD outbreak worldwide, according to the World Health Organization (WHO), since its discovery in 1967 [8,9]. In Africa, MVD outbreaks have been reported in Angola, the Democratic Republic of Congo (DRC), South Africa, Equatorial Guinea, Uganda, Kenya, and a recent outbreak in Rwanda [3,6,10,11].

Tanzania is at potential risk for MVD outbreaks due to its diverse ecosystem, including tropical rainforests and caves, which provide suitable habitats for fruit bats that are reservoirs for MVD [7,12,13]. The presence of human interactions due to agriculture and economic activities with wildlife, including bats, increases the risk of zoonotic spillover. Additionally, the abundant wildlife in Tanzania, including primates like chimpanzees, can serve as reservoirs for the MVD virus, further elevating the risk [7,12,13]. Tanzania’s proximity to countries with reported MVD outbreaks, including Uganda and the Congo DRC, also serves as a risk factor for MVD outbreaks through cross-border transmission risk [14].

In response to the outbreak declaration, the Tanzania MoH activated the incident management system (IMS) and established multiple response pillars, including surveillance [15]. The Kagera regional surveillance coordinator led the surveillance pillar, comprising national and district surveillance teams and partners.

### EBS implementation in Tanzania

**Fig 1:**
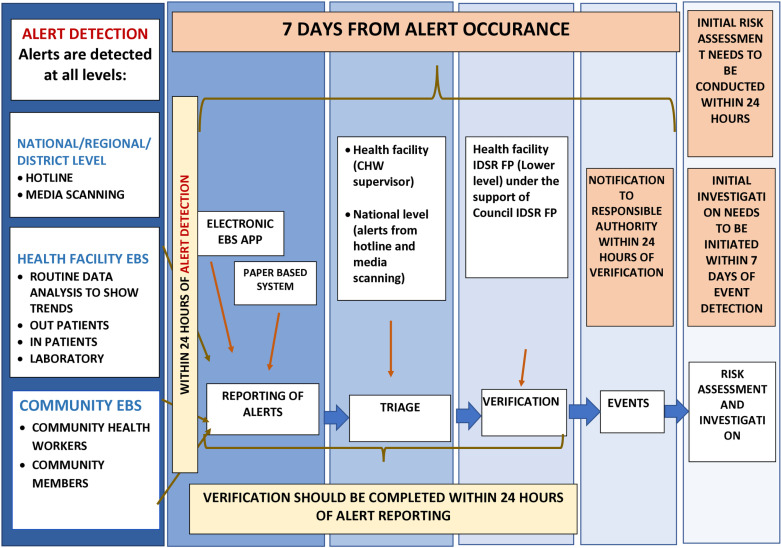
Signal management flow and the steps for the implementation of EBS in Tanzania.

Referring to Fig 1, Tanzania EWAR relies on EBS to detect and immediately report signals. EBS enables early detection of outbreaks and other events of public health importance. EBS operates across multiple levels, including community, health facilities, councils, regions and national levels [16]. The initial step for EBS begins with the detection of priority signals, referred to as any information or patterns of disease identified by the EWAR as the potential acute risk to human health, such as an outbreak [16–18]. To facilitate easy detection of signals by CHWs and HCWs, the MoH developed a simple (layman’s) list of priority signals at both the community level and healthcare facilities [16]. Detected signals are then reported physically to a health facility, the MoH Afya Call Center (hotline number 199), the electronic EBS application (e-EBS App), WhatsApp groups, and dedicated regional and council EBS desk numbers. The e-EBS app, available as a mobile- and web-based digital tool, facilitates timely reporting and monitoring of public health events (PHEs) at all levels. The app provides a guided process for reporting signals, making it easier to use and ensuring consistency. Signals reported via the 199-hotline number are directly integrated into the e-EBS app. The signals reported through WhatsApp groups and dedicated regional and council EBS desk numbers must be manually entered into the e-EBS app. To facilitate rapid detection of signals, CHWs conduct community sensitization through various gatherings such as business hotspots, food vendors, open markets, and village meetings, including at health facilities, though limited resources restrict the reach of these sites.

HCWs triage all reported signals under the supervision of the council IDSR FP [16–18]. Signals are triaged to ensure the information received is of public health importance and not duplicative of previous reports. The e-EBS app facilitates the recording of signal management steps and provides a platform for higher-level management to visualize and effectively manage signals. All triaged signals are then verified physically at the locality or by a phone call to a trusted source to determine if they have truly happened. True verified signals are then evaluated using the standard case definition (SCD) for confirmation if they are events indicating a PHE that need notification or alerting for initiating response efforts, including risk assessment, within 48 hours of confirmation, as outlined in the national IDSR third edition guideline [17]. The IDSR guideline includes SCDs for viral hemorrhagic fever (VHFs), including MVD [17]. True verified signals not meeting the SCD criteria are managed at a health facility level. Continuous training for surveillance officers, HCWs, and CHWs ensures that they report unusual signals, even if these signals do not align with existing criteria (Fig 1). The e-EBS platform allows real-time access to reported signals at all levels, ensuring timely responses and follow-ups. Signal reporting must be completed within 24 hours of detection, and subsequent triage and verification must be conducted within 24 hours (Fig 1).

Tanzania also uses the 7-1-7 target to measure the timeliness of detection, notification, and response to public health threats [19]. Events must be detected within seven days of occurrence, relevant authorities must be notified within one day, and seven days to complete initial response efforts [19].

### EBS implementation in the Kagera region before the outbreak

Since 2020, the Tanzania MoH, in collaboration with the President’s Office, Regional Administration, and Local Government of Tanzania (PO-RALG), in partnership with Amref Health Africa-Tanzania and the U.S. Centers for Disease Control and Prevention (CDC), through the Global Health Security Agenda (GHSA) project, has established EBS implementation in the Kagera region. This partnership strengthened capacity through training, mentorship, and supervision for CHWs and HCWs on detecting, reporting, and verifying signals, and raising awareness among influential, administrative, and religious leaders. By March 2023, the training had reached 740 CHWs and 310 HCWs across all eight districts of the Kagera region. Additionally, 18 (2 regional and 16 district councils) IDSR FPs were capacitated on EBS data management.

Following the September 2022 EVD outbreak in Uganda, 90 CHWs and 46 HCWs in the Kagera region underwent e-EBS training by the MoH. The training was part of the VHF operational readiness to strengthen EWAR initiatives in four districts [Kyerwa (45), Muleba (15), Missenyi (20), and Bukoba DC (12)]. CHWs received smartphones to support electronic signal reporting from the community.

### Description of the outbreak area

Kagera region is the northwestern part of the country, with a population of 2,989,299, according to the 2022 national census [20]. It is bordered by Lake Victoria, Mwanza, and Mara Region to the east, Geita Region and Burundi to the south, Kigoma Region and Rwanda to the west, and Uganda to the north. Because it is a transport corridor to Uganda, the Congo DRC, and other parts of central Africa, it is considered a high-risk area for cross-border disease transmission [21,22].

The region’s capital city is the Bukoba district, where the outbreak occurred. It is one of the seven districts in the Kagera region, divided into 28 wards [2]. It is bordered by the Missenyi district to the north, Lake Victoria to the east, the Muleba district to the south, and the Karagwe district to the west. The population of the Bukoba district is 322,448, of which 156,788 are males and 165,660 are females, as reported by the Tanzania National Census of 2022 [20]. The district has a total of 81,859 households with an average household size of 3.9. The main economic activities include agriculture, petty trade, and fishing. Rainfall varies from 500 mm to 2000 mm per year [20]. The temperature ranges from 20 to 28 degrees Celsius. A major paved road from Mwanza to the Uganda border passes through Bukoba district [21,22].

The health administration system of the Kagera region consists of the District Medical Officer (DMO) for each of the seven districts reporting to the Regional Medical Officer (RMO), who then reports to the Chief Medical Officer (CMO) at the MoH. The region has almost 800 CHWs who, together with other functions assigned, support the implementation of EBS at the community level. Bukoba district has 21 registered health facilities, including dispensaries, health centres, and district hospitals.

### Reporting of the first MVD signal

The probable index case, a 33-year-old man, a fisherman from the Bukoba district, began experiencing symptoms of fever, generalized body weakness, and later bleeding from body orifices on February 27, 2023. He initially received home care from his relatives, but his condition worsened as he started bleeding from his body openings. On February 28, 2023, a motorcycle driver took him to a local health facility where a rapid test for malaria was performed. Due to the severity of his condition, he was referred to a regional referral hospital (RRH) on March 1, 2023, but died on the way to the hospital [23]. As MVD was not suspected, the deceased was given an unsupervised burial on March 2, 2023.

Subsequently, on March 8, 2023, two other relatives showed similar signs and symptoms and sought health care at different health facilities using the same motorcycle driver from the community. By March 12, 2023, two additional people became sick, and five died after displaying similar signs and symptoms of an unknown disease. By March 15, 2023, there was a raised fear in the community due to the occurrence of five alarming deaths from individuals who displayed similar signs and symptoms within a short period. On March 15, 2023, a previously mentioned motorcycle driver who transported four patients, including the index case, to different health facilities informed a community leader in the affected ward. The community leader, in turn, notified the DMO, who confirmed the signal to be an event through district surveillance officers and health facilities. Simultaneously, a trained CHW reported a similar signal into the e-EBS application on the same date within 24 hours of signal detection from the ongoing signal, leading to an automatic notification to MoH. Subsequently, the RMO was informed on the same date (March 15, 2024), and on March 16, 2023, he notified the MoH.

Following these initial reports, the initial investigation was carried out by district and regional rapid response teams (RRTs) and consequent laboratory sampling by the national RRTs, where laboratory confirmation was completed at a local mobile public health laboratory by March 19, 2023 (4 days), leading to the declaration of the MVD outbreak. This declaration triggered the action of the Emergency Operation Center and the IMS response. In the surveillance pillar, EBS interventions included strengthening the EBS desk for signal reporting and community sensitization. By the outbreak’s end, eight samples tested positive for the MVD.

After nearly three months of response efforts by the government and other partners, the outbreak’s end was declared 42 days later, on May 31, 2023 [24], after the last confirmed case on April 11, 2023.

This study documents the EWAR interventions carried out through EBS implementation during the MVD outbreak response in the Kagera region. The aim is to document the EWAR system’s structure, processes, and performance in detecting, reporting, and monitoring MVD signals and cases to identify strengths and bottlenecks that may facilitate or hinder timely outbreak detection and response. The findings will provide recommendations to strengthen future outbreak preparedness and response efforts for immediate detection, reporting and control of outbreaks.

## Method

We documented EWAR interventions using a retrospective surveillance document review of the signal data reported before the declaration and during the outbreak response. Surveillance interventions included assessing outbreak timeliness using Tanzania’s EBS indicators and the 7-1-7 target for detection, notification, and response; effectiveness strengthening the EBS desk for monitoring signal detection and reporting; community sensitization; and EBS data management.

### Measurement of outbreak time metric for EWAR

**Fig 2:**
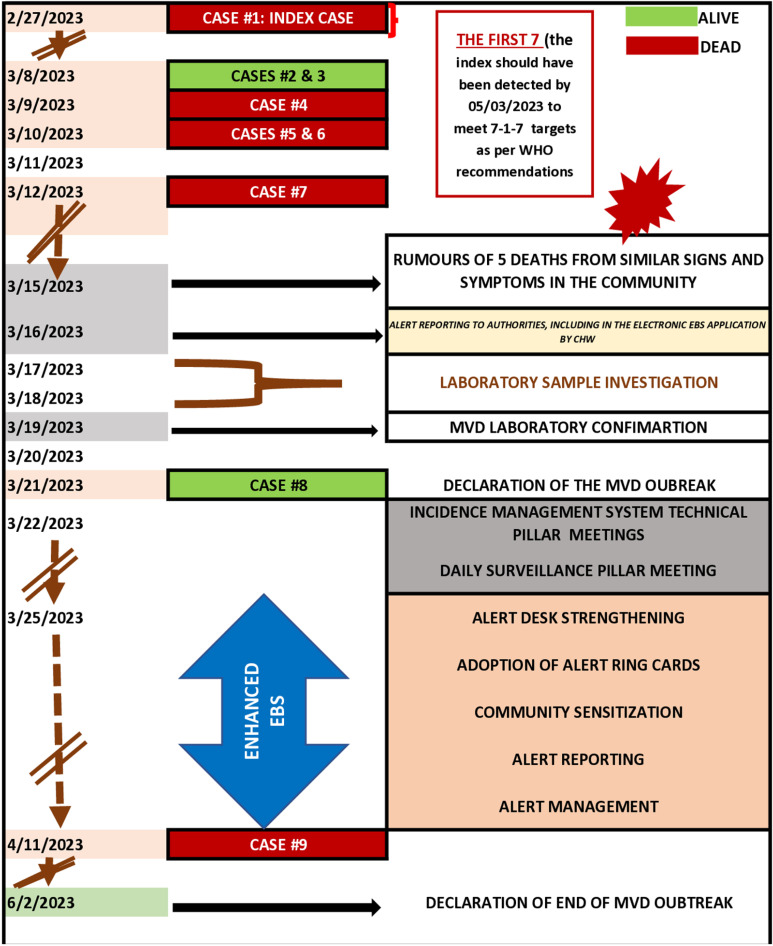
Time metrics during the MVD outbreak response with EBS interventions.

This study used the 7-1-7 metrics to help measure the timeliness associated with this outbreak. The assessment covered key events from the onset of symptoms in the index case to the official declaration of the outbreak’s end (Fig 2). Timeliness was calculated by counting the days between key outbreak events. The first “7” of the 7-1-7 target was evaluated by calculating the duration from the onset of symptoms in the index case to the event detection. Outbreak detection was considered “timely” if detected within seven days of symptom onset. The time to notification was measured from the detection of the event to its official reporting to authorities. If notification occurred within 24 hours of detection, it was categorized as “early notification”. The time to initial response was determined by measuring the time from the notification to authorities to the initiation of response action, including RRT deployment and laboratory investigation for event confirmation. If the initial response occurred within seven days, it was classified as an “early initial response”. In addition, Tanzania EBS indicators complemented the 7-1-7 metrics. At the community level, the timeliness was measured by calculating the duration from the emergence of a signal to its reporting in the formal reporting channels, including eEBS. At the health facility level, the timeliness was calculated by measuring the duration from when the index case was first presented to the health facility to when it was recognized by HCWs and reported as a potential PHE. The bottleneck assessment discussion was employed to understand why the process performed well or was delayed, and how to improve it.

### Strengthening of EBS desk

In response to the MVD outbreak, a rapid situational assessment was conducted to assess the surveillance capacity for MVD response. Following this, actions to strengthen the surveillance team included deploying additional surveillance officers from the MoH and other implementing partners, equipment and adoption tools. Staff received orientation on MVD response protocols. Terms of reference and essential equipment, such as computers, phones, and airtime, were supplied for deployed staff to enhance communication and signal data management. The surveillance team received signals from the community through multiple channels.

Adapted tools included informational cards from the MoH’s RING cards (Recognize, Isolate, Notify, and Get-help) approach used at points of entry (PoE) to screen ill travelers. These durable, pocket-sized cards, printed on durable Manila paper, displayed MVD signs and symptoms, regional EBS desk numbers, the national toll-free number (199), and clear instructions for reporting procedures. The signs and symptoms were translated into Swahili, the commonly used language in the outbreak area, to enhance awareness further. The symptoms listed on the informational cards included fever, headache, vomiting, nausea, diarrhea, abdominal pain, loss of appetite, generalized body weakness, joint pain, muscle ache, bleeding from body openings, body rash, cough, sore throat, difficulty breathing, hiccups, convulsions, and sudden death from an unknown cause.

### Community sensitization for awareness creation on signal reporting

A situational assessment was conducted to develop a sensitization plan to target high-risk groups. Key stakeholders who conducted sensitization included surveillance officers, local health authorities like village local leaders, and CHWs in collaboration with Risk Communication and Community Engagement (RCCE) teams to ensure effective coordination. Informational cards were distributed to the community during community sensitization gatherings.

### Data collection

Data used in this study were extracted from the signal data collected during the outbreak from March to May 2023, before and after the declaration. Reported signals were compiled in Microsoft Excel (version 2019), developed specifically for monitoring signal reporting for the current outbreak response. The template included key variables such as the date and time of signal detection, detailed location information (region, district, and street/village), and the signal source (e.g., CHWs, HCWs, or other community members). Additional variables included clinical signs and symptoms, such as whether the case met the SCD, or two or more layman’s MVD signs and symptoms displayed in the informational cards. Laboratory details were also included, such as the date and time of sample collection and the corresponding results.

The documentation of time metrics and effectiveness of EBS implementation utilized signal data collected and recorded retrospectively and during the outbreak period, before and after the declaration, covering timeliness from the onset of symptoms in the index case to the official declaration of the outbreak’s end. All key outbreak events were systematically recorded in Microsoft Excel to ensure consistency and accuracy. Data were cross-checked for validation. The time metrics for detection, notification, and initial response were calculated by measuring the duration between key outbreak events to assess timeliness and identify delays.

### Data management and analysis

The signal management process began with compiling signals into the Excel spreadsheet, followed by cleaning to remove duplicates and correct inconsistencies. Signals were analyzed using descriptive statistics in Microsoft Excel. Frequency analysis to show trends across various variables, including time (monthly), geographic distribution, and presenting signs and symptoms.

### Ethical considerations

The information presented in this manuscript was collected during the MVD outbreak response, conducted as part of an emergency public health activity under the supervision and approval of MoH. Formal ethical approval was not required because the investigation was carried out under emergency circumstances. However, the Medical Research Coordinating Committee (MRCC) of the National Institute for Medical Research (NIMR) granted a waiver for data analysis and permission to publish the findings. All personal information collected during the investigation was handled with the highest level of confidentiality, ensuring adherence to established data protection standards. Identifiable information was excluded from the analysis, and the data presented in this manuscript have been fully anonymized to protect the privacy of individuals.

## Results

In this study, signal detection occurred 16 days after the onset of symptoms in a probable index case. At the community level, there was a missed opportunity for early recognition of the signal, as the motorcycle driver who transported the index and other relatives to the health facility during the initial visit did not identify or report the unusual signs and symptoms of MVD. Similarly, at the health facility, HCWs failed to detect the unusual signs during the MVD index case’s first visit. However, once the signal emerged in the community, the CHWs reported the signal in the e-EBS. An automatic notification was sent to the national, regional and council authorities within 24 hours.

The initial response started on March 16, 2024, within one day of notification, which established an epidemiological link to a probable index case who had died on March 1, 2023, three days after the onset of symptoms. Laboratory confirmation of MVD was obtained on March 19, 2023, twenty (20) days after the onset of symptoms of the index case, leading to the outbreak’s official declaration on March 21, 2023. The outbreak was contained by May 31, 2023, following nearly 78 days of response (Fig 4).

**Fig 3:**
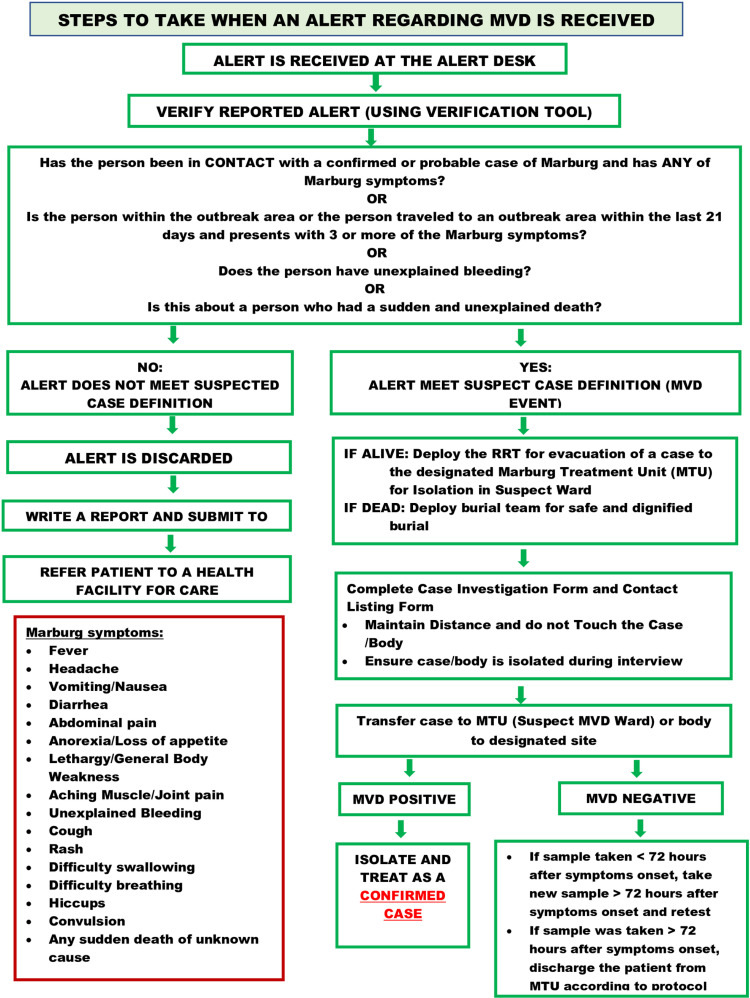
Signal verification tool for MVD used by MOH Tanzania, March 2023.

**Fig 4:**
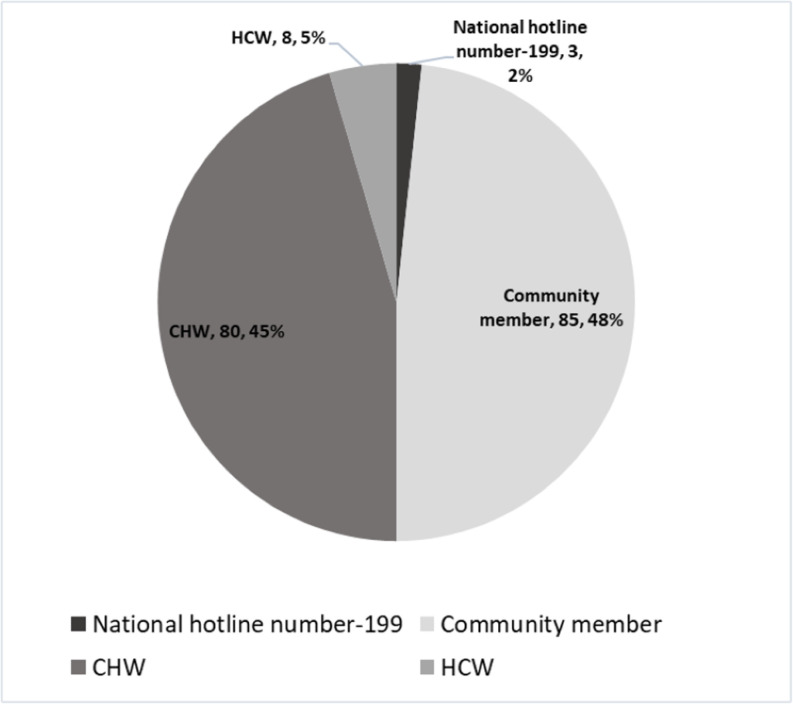
Signals captured from different sources in the Kagera region, Tanzania, during MVD response from March 21 to May 31, 2023.

Five (5) additional surveillance officers were deployed and mentored on signal reporting procedures to facilitate MVD data management, making a total of ten (10) officers at the EBS desk. To further strengthen the EBS desk, information, communication, and technology (ICT) equipment (10 smartphones and three laptops) were distributed to surveillance officers.

Between March 21 and May 31, 2023, 6,260 informational cards were produced and disseminated during sensitization meetings in the Bukoba district and nearby regions. The sessions were conducted daily in multiple high-risk areas, including churches, mosques, markets, offices, fishing camps, motorcycle stations, hotels, and clubs. Additionally, informational cards were distributed to pupils and teachers in schools. MVD information was disseminated through local radios and the public announcement system (PA). The CHWs also supported disseminating MVD messages in village meetings and through door-to-door house visits. The surveillance officers verified the signal reported using an adopted tool (Fig 3).

Signal desk strengthening and community sensitization efforts led to the detecting and verifying of 176 MVD signals during the same period. The distribution of signals according to reporting sources is represented in Fig 4. The sources of signals were from CHWs, HCWs, and community members. Most of the signals came from community members 88 (50.0%), followed by 80 (45.5%) from the CHWs. Bukoba district accounted for the most signals, with 74 (38.5%).

The most significant surge in signals reported during the implementation of surveillance intervention was in April 2023, with 107 (60.8%), an increase of 613.3% as compared to 15 (8.5%) in March and 54 (30.7%) in May 2023. The most commonly reported symptoms were fever 56 (31.8%), headache 53 (30.1%), diarrhea 27 (15.3%), bloody diarrhea 26 (14.8%) and abdominal pain 22 (14.2%) (Fig 4).

Of the 176 verified signals, 48 (27.3%) met MVD SCD and were submitted to the RRT for further investigation and laboratory confirmation. A total of 37 (77.1%) were sampled and subjected to laboratory confirmation using reverse-transcriptase polymerase chain reaction (RT-PCR). Of the laboratory confirmed, one (2.7%) turned out positive.

Monitoring of signal reporting was conducted in surveillance pillar meetings to improve the performance of reporting and management.

## Discussion

This paper documents EBS interventions to strengthen EWAR during the MVD outbreak response. These activities include adopting, printing, and disseminating MVD informational cards, community sensitization, and strengthening EBS desks to improve signal management. It also evaluated the timeliness of signal reporting and management, the effectiveness of EBS implementation, and the MVD surveillance response in the Kagera region.

In this study, there was a prompt reporting of MVD signals within 24 hours of detection by CHW through electronic EBS, despite the initial delay in detecting the index case by a community member and HCWs. The e-EBS facilitated the real-time information on emerging MVD cases which might be due to the e-EBS retraining that these sites received. A report by Lombardo et al. demonstrated that e-EBS facilitates the timely response to outbreaks [25]. A prompt signal reporting from the CHW trained in e-EBS indicates the effectiveness of using e-EBS to facilitate rapid reporting and detection of the outbreak which delineates the path for enhancing its effectiveness of signal reporting and EWAR.

In the current study, there was a three-week delay in recognizing the probable index case at health facilities. The delayed signal recognition likely contributed to outbreak’s possible spread 6to close family members. Similar to the current outbreak, delays in outbreak detection have been reported in Ebola outbreaks in Congo DRC in 2007 (90 days) and 2018 (92 days) and in West Africa in 2013–2016 (86 days) [26]. Another paper describing lessons learned during the EVD Outbreak in Lofa County, Liberia, in 2014 cites the primary reason for delays was the absence of surveillance and early warning systems to report the occurrence of unusual events immediately [27]. Effective EWAR and surveillance in health facilities proves to be effective in early detection of outbreak as results from the systematic review by Meckawy R. et al., who reported that EWAR can function successfully and independently as surveillance systems for detecting pandemic-scale infectious disease outbreaks [22].

The bottleneck analysis of the current study identified multiple challenges in the detection and reporting of the MVD outbreak, both at the community and health facility levels. During the initial visit of the probable index case to a health facility, the patient exhibited symptoms but was only tested for malaria and referred to another facility. The incident resulted in a missed opportunity to suspect VHFs. The current delay can be attributed to the inadequate surveillance and EWAR system by community members and HCWs on epidemic-prone diseases, although more studies are needed. Matson MJ et al. on delayed recognition of the Ebola virus 2020 indicate that numerous differential diagnoses of Ebola resulted in a low suspicious rate similar to the current outbreak [26].

However, unlike the findings of this study, research by Chaudhry A. et al. on the effectiveness of EBS in signal detection highlighted that health facility-based EBS was able to identify vector-borne diseases and other conditions, indicating its significant contributions in outbreak detection [28].

Additionally, the community member, a motorcycle driver transported the probable index case to the health facility but did not recognize the unusual symptoms, leading to a missed opportunity for early detection. This scenario is similar to the incident during the 2017 EVD outbreak in the DRC, where a motorcycle driver transported a patient to a health facility. The patient later died and was identified as a probable case of EVD [29]. Tanzania’s new IDSR guidelines recommend strengthening community capacity, particularly in high-risk areas, to ensure early detection of PHEs [16]. Before the strengthening of EBS interventions, low reporting signals can be attributed to insufficient sensitization to EWAR, especially in high-risk regions.

In our current study, CHWs were engaged in response intervention to facilitate signal reporting from the community. CHW engagement intervention was also reported in several studies during the COVID-19 and Ebola outbreaks [30,31]. During the MVD outbreak, CHWs distributed informational cards and provided MVD health education, including detecting, preventing, and reporting signals [32]. The engagement indicates a multisectoral approach to surveillance outbreak response efforts. Adapted tools, including signal tools as a quick reference sheet for clinics and community members, were implemented to create awareness and increase signal reporting. Signal reporting increased in April compared to the prior month and could be attributed to enhanced community sensitization. Similar interventions have been reported in a paper by Oleribe O.O. et al. on the EVD outbreak in West Africa, where community sensitization has facilitated early outbreak containment. Community sensitization was also encouraged by strong collaboration with other pillars, including RCCE, reaching a wide coverage and increasing the community’s signal reporting.

Laboratory results from the reported signals confirmed only one (2.7%) positive case among the 37 samples sent for testing. The current study’s findings differ from those of Christopher H. Hsu et al., who conducted research in West Africa between 2016 and 2018 and found that nearly 45.8% of cases that met the case definition were confirmed for Ebola [33]. The discrepancy in findings may be explained by the short duration of the outbreak that affected close family members [23]. Of the positive cases reported through the MVD outbreak, seven (77.8%) out of nine confirmed cases were reported during the initial stages before EBS interventions started on March 17, 2024, which could reflect the effectiveness of control measures in rapidly halting transmission.

## Conclusion

The current study highlighted the EWAR response interventions carried out during the MVD outbreak in Kagera from March to May 2023. This study observed timely reporting of MVD signal, however, there was a delay in the detection of the outbreak. The government and stakeholders should adopt the 7-1-7 target as mandated by the WHO and strengthen community and health facility EBS through ongoing mentorship for early detection, notification and response to MVD outbreak. Additionally, equipping surveillance staff with adequate surveillance tools is essential for EWAR.
